# Myeloperoxidase impairs mucociliary transport on human airway epithelium

**DOI:** 10.1242/dmm.052764

**Published:** 2026-05-26

**Authors:** Allison Boboltz, Vaidehi Rathi, Sahana Kumar, Gregg A. Duncan

**Affiliations:** ^1^ Fischell Department of Bioengineering, University of Maryland, College Park, MD 20742, USA; ^2^ Biological Sciences Graduate Program, University of Maryland, College Park, MD 20742, USA

**Keywords:** Myeloperoxidase, Airway clearance, Mucus, Mucolytics

## Abstract

Dampening neutrophil-driven inflammation in the airways remains a challenge in treating cystic fibrosis (CF) lung disease. Myeloperoxidase (MPO) is a neutrophilic enzyme that produces reactive oxygen species and is highly concentrated in CF airways. Greater MPO concentrations have previously been correlated with increased mucus plugging in bronchiectasis, suggesting that MPO could impair mucociliary transport. As such, we evaluated the impact of MPO treatment on barrier integrity, mucin production, mucus viscoelasticity and mucociliary transport in fully differentiated human airway epithelial cultures at ionic conditions reflective of healthy and CF-affected airways. Using live-cell imaging and particle velocimetry, we found that MPO inhibits mucociliary transport *in vitro* at CF-like and normal airway conditions. The impairment of mucus clearance by MPO was similar to that by neutrophil elastase, another neutrophilic granular enzyme that damages the host tissues and impairs airway clearance. We also found that subsequent treatment with the reducing agent, N-acetyl cysteine, could alleviate MPO-mediated mucociliary dysfunction through disulfide bond cleavage. These findings identify MPO as a therapeutic target to resolve deficits in airway clearance function in CF and related muco-obstructive lung diseases.

## Teaser

**Summary:** Myeloperoxidase, a neutrophilic granular protein found abundantly in the lungs in cystic fibrosis, was found to directly inhibit airway clearance function and may represent a new therapeutic target for cystic fibrosis.

## INTRODUCTION

Cystic fibrosis (CF) is an incurable genetic disorder caused by variants in the *CFTR* gene that result in malfunctioning of the cystic fibrosis transmembrane conductance regulator protein (CFTR) (Grasemann and Ratjen, 2023). In the lungs, CFTR controls the ionic composition of the airway surface layer by transporting anions such as chloride (Cl^−^) and thiocyanate (SCN^−^) (Xu et al., 2009). Maintaining proper hydration and ionic gradients in the airway surface layer are important in supporting normal mucociliary transport (MCT) (Song et al., 2020; Boucher, 2019). During MCT, mucus is cleared by airway epithelium via the coordinated beating of cilia in the periciliary layer. Mucus forms a protective gel barrier lining the airways to trap inhaled particles and microbes before being removed from the airways via MCT. Mucus consists of ∼98% water and ∼2% solids in health (Boucher, 2019). The solid components of mucus include various proteins such as mucin glycoproteins and DNA from dying airway epithelial and immune cells. In CF, CFTR dysfunction leads to highly viscoelastic mucus with an increased percentage of solid components (often >10% solids) that is not effectively cleared by MCT (Boucher, 2019). Another downstream effect of the thick, stagnant mucus is uncontrolled inflammation in CF airways. Inhaled bacteria that become trapped in the dense mucus can colonize the airways and form biofilms that lead to chronic infections, causing inflammation. In addition, the viscous mucus layer can cause hypoxia-induced necrosis of the airway epithelial cells, leading to ‘sterile inflammation’ with no infection (Montgomery et al., 2017). The excess release of inflammatory mediators in CF airways continues to promote the secretion of various proteins and release of DNA, which further enhance the viscoelasticity of the mucus in a cyclic manner.

The inflammatory response in CF is dominated by neutrophils that migrate across the bronchial epithelium into the mucosal barrier. Neutrophil effector functions fall into three main subcategories: degranulation, neutrophil extracellular trap release (NETosis) or phagocytosis (Rosales, 2018). All neutrophil effector functions involve the use of granules containing enzymes including myeloperoxidase (MPO), which is one of the most abundant proteins in neutrophils (Schultz and Kaminker, 1962). Both degranulation and NETosis involve the extracellular release of MPO, causing elevated levels in CF airways (Kettle et al., 2004; Van der Vliet et al., 2000). Whereas degranulation releases free MPO, NETosis releases MPO that is complexed with DNA from de-condensed chromatin and greatly reduces its enzymatic activity (Worlitzsch et al., 1998; Parker et al., 2012). However, a large population of people with CF inhale DNase daily to degrade the chromatin structure of neutrophil extracellular traps (NETs), which release active MPO (Parker et al., 2012). MPO reacts with the substrates hydrogen peroxide (H_2_O_2_) and halide or pseudohalide ions, including SCN^−^ and Cl^−^, to produce the oxidant acids hypothiocyanous acid (HOSCN) and hypochlorous acid (HOCl), respectively (van Dalen et al., 1997). H_2_O_2_ is secreted by the airway epithelium and activated neutrophils, and both the Cl^−^ and SCN^−^ substrates are transported by CFTR into the airway surface layer (Fragoso et al., 2004; Wright et al., 1994). Although these highly potent oxidant acids are very effective at killing microbial pathogens, damage to the host tissues can also occur. MPO treatment has been shown to cause cell death of human airway epithelial (HAE) cells in submerged culture via oxidative stress (Xu et al., 2009; Kim et al., 2023; Van der Vliet et al., 2000). In addition, these studies have found that the addition of SCN^−^ and subsequent generation of HOSCN reduce cytotoxicity dramatically in comparison to that from HOCl, which is highly cytotoxic (Xu et al., 2009; Kim et al., 2023).

SCN^−^ is drastically reduced in CF airways owing to CFTR malfunction, potentially leading to worsened damage, with increased generation of HOCl over HOSCN (Van der Vliet et al., 2000; Bihler et al., 2024; Malkovskiy et al., 2019). CFTR functional rescue by modulator drugs may increase the concentration of SCN^−^ in the airways, increasing the generation of HOSCN by MPO and leading to potential differences in mucociliary phenotypes. CFTR modulator medications, especially the highly effective triple combination therapy elexacaftor/tezacaftor/ivacaftor (ETI), provide ≥10% improvements in CFTR function *in vitro* (Bihler et al., 2024). Although the effects of CFTR modulators on SCN^−^ concentration specifically in sputum samples has not been evaluated, a study that characterized SCN^−^ in the saliva of people with CF found that samples taken from people receiving single CFTR modulator therapies (such as ivacaftor, lumacaftor or tezacaftor) had a significantly higher amount of SCN^−^ than did those not taking modulators (Malkovskiy et al., 2019). It is likely with the introduction of the highly effective triple combination modulator therapy ETI that the amount of SCN^−^ in the airways is even further increased. Although ∼90% of the CF population is eligible for ETI, it remains inaccessible to a large portion of people with CF globally. A 2024 study estimated that only 27% of people with CF worldwide were receiving triple combination therapy (Guo et al., 2024). Furthermore, in the United States, ∼8% of people with CF are ineligible for CFTR modulators owing to incompatibility with their genetic variant, and another ∼10% of people eligible for CFTR modulators are not using modulators owing to various factors such as drug side effects (Kramer-Golinkoff et al., 2022; Cystic Fibrosis Foundation Patient Registry, 2025). Given the disparities in access to modulator therapies, continued research into potential targets for anti-inflammatory treatments for those receiving and not receiving CFTR modulator therapies is merited.

Despite the potential clinical implications of increased MPO in causing oxidative stress and worsening of CF lung disease (Dickerhof et al., 2020), it remains largely unknown how MPO affects MCT in the airways. Previous research found that MPO concentrations in people with non-CF bronchiectasis were correlated with more mucus plugging in patients, indicating, presumably, a lack of effective MCT (Kim et al., 2025). Prior *in vitro* studies evaluating the impacts of MPO on the airways have relied on cell lines that do not generate their own mucus and/or cilia, making evaluation of MCT impossible. Motivated by this, we used an *in vitro* model of the airway epithelium to study how MPO in the mucosal barrier affects mucociliary phenotypes. Specifically, we conducted these studies in BCi-NS1.1 human airway epithelial cultures fully differentiated at air–liquid interface (ALI), which possess otherwise normal MCT function. We assessed these cultures after the exogenous addition of MPO in normal and CF-like conditions to determine their impact on MCT and airway surface liquid composition. Ultimately, these findings strengthen the rationale for therapeutically targeting MPO in CF and other muco-obstructive lung diseases.

## RESULTS

**Fig. 1.**
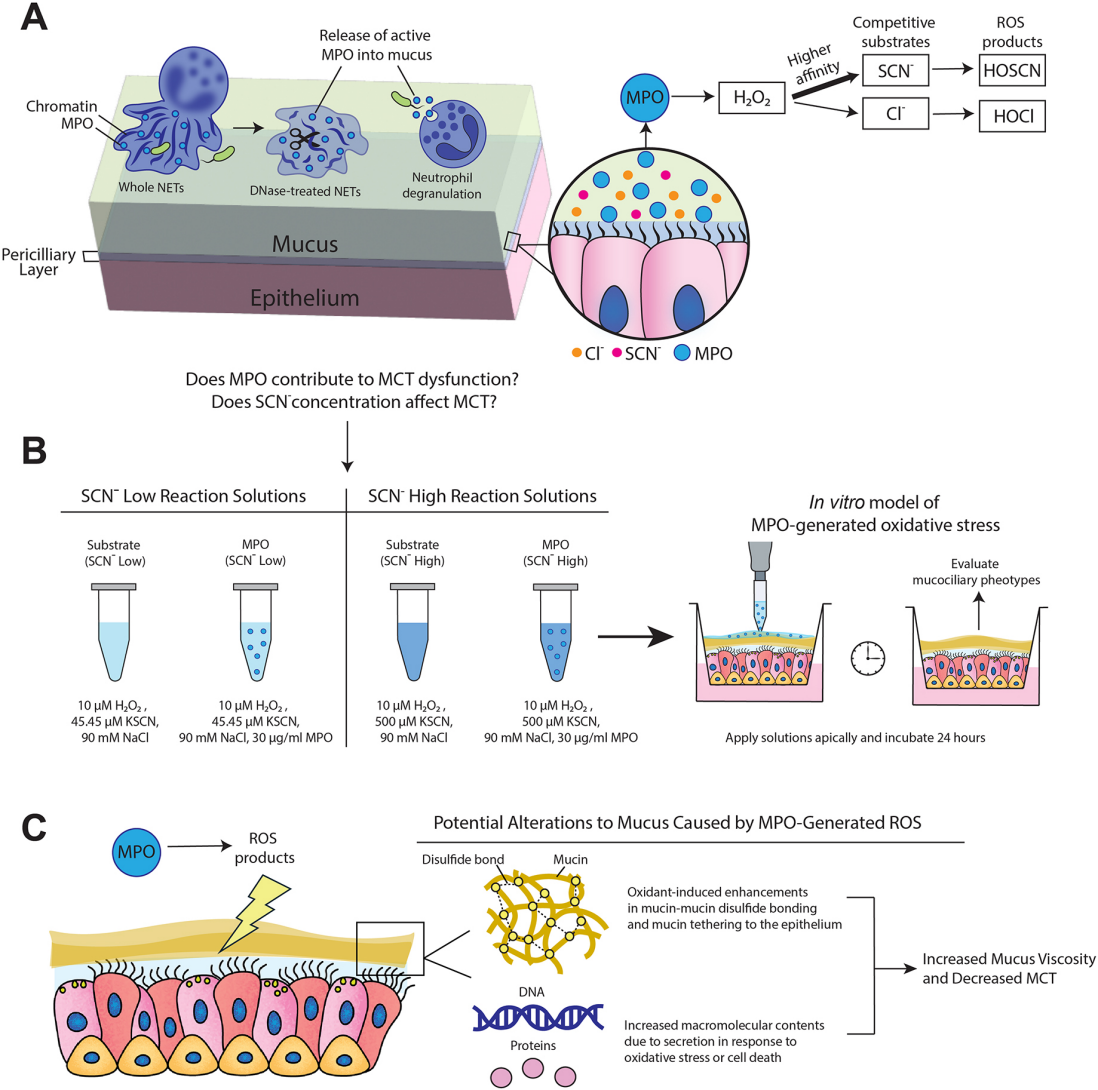
**Modeling cystic fibrosis (CF)-like conditions *in vitro* to study the impact of myeloperoxidase (MPO) on mucociliary function.** (A) Schematic showing how neutrophilic inflammation in the airways leads to increased MPO concentrations in the mucus via degranulation and neutrophil extracellular trap release (NETosis), and how MPO reacts with substrates in the mucus layer to form hypothiocyanous acid (HOSCN) and hypochlorous acid (HOCl) reactive oxygen species (ROS). NET, neutrophil extracellular trap. (B) Schematic showing the *in vitro* model created to address the central research questions of this work concerning the effects of MPO on mucociliary transport (MCT) and the role of thiocyanate (SCN^−^) concentration in altering mucociliary phenotypes. The SCN^−^ concentration was altered by creating two different substrate solutions, SCN^−^ low and SCN^−^ high. Four different treatment groups were established using the SCN^−^ low and SCN^−^ high substrate solutions, two of which contained MPO and two that consisted of the substrates alone. The substrate-only or MPO–substrate solutions were applied apically to the surface of human airway epithelial (HAE) cultures and incubated for 24 h. (C) Schematic of potential alterations to the mucus upon exposure of the airway epithelium to MPO-generated ROS, which will be investigated in this work. We hypothesize that exposure to ROS will increase the viscosity of mucus and reduce MCT. Cl^−^, chloride; H_2_O_2_, hydrogen peroxide; KSCN, potassium thiocyanate; NaCl, sodium chloride.

Free MPO is released into the CF mucosal barrier via neutrophil degranulation and/or the degradation of NETs by DNase inhalation (Fig. 1A). In designing our *in vitro* model to study how MPO affects mucociliary phenotypes, we created two different substrate solutions to react with MPO, each containing H_2_O_2_, SCN^−^ and Cl^−^ at concentrations relevant to the CF airway surface layer (Fig. 1B) (Yuan et al., 2015; Zabner et al., 1998; Widdicombe, 1999; Jayaraman et al., 2001). The only variable between the two substrate solutions was the concentration of SCN^−^. The substrate solution designated as ‘SCN^−^ high’ contained the normal concentration of SCN^−^ ions found in the airway surface layer (500 μM), and the ‘SCN^−^ low’ substrate solution contained 11 times less SCN^−^ (45.45 μM) to mimic the concentration in CF airways without CFTR modulators (Kettle et al., 2004; Wijkstrom-Frei et al., 2003; Conner et al., 2007). The estimate of 45.45 μM was based upon a study using HAE cell cultures from CF donors grown at ALI that found that 11 times less SCN^−^ was transported from the media in the basolateral chamber into the apical airway surface layer than in HAE cell cultures from unaffected controls (Conner et al., 2007). Based on the specificity constants, we calculated the theoretical ratio of HOSCN to HOCl production by MPO in the SCN^−^ high solution to be ∼27% HOSCN to ∼73% HOCl. In the SCN^−^ low solution, this ratio is calculated to be ∼80% HOSCN to ∼20% HOCl (Van Dalen et al., 1997). We combined the substrate solutions with 30 μg/ml MPO, representative of the average concentration reported in CF sputum samples (Yuan et al., 2015; Dittrich et al., 2018). To evaluate the specific contributions of MPO to mucociliary dysfunction, we also applied both the SCN^−^ high and SCN^−^ low substrate solutions with no MPO added. We applied a 20 μl volume of the MPO–substrate or substrate-only solutions to cover the apical surface and allowed the cultures to incubate for 24 h (Fig. 1B). During this time, the small volume of liquid originally added to the airway surface layer was absorbed, and mucociliary function could subsequently be evaluated. We investigated several different mechanisms by which MPO-generated reactive oxygen species (ROS) may affect MCT by damaging the epithelium and altering the structure or composition of the mucus gel (Fig. 1C).

### MPO reduces MCT *in vitro*

**Fig. 2.**
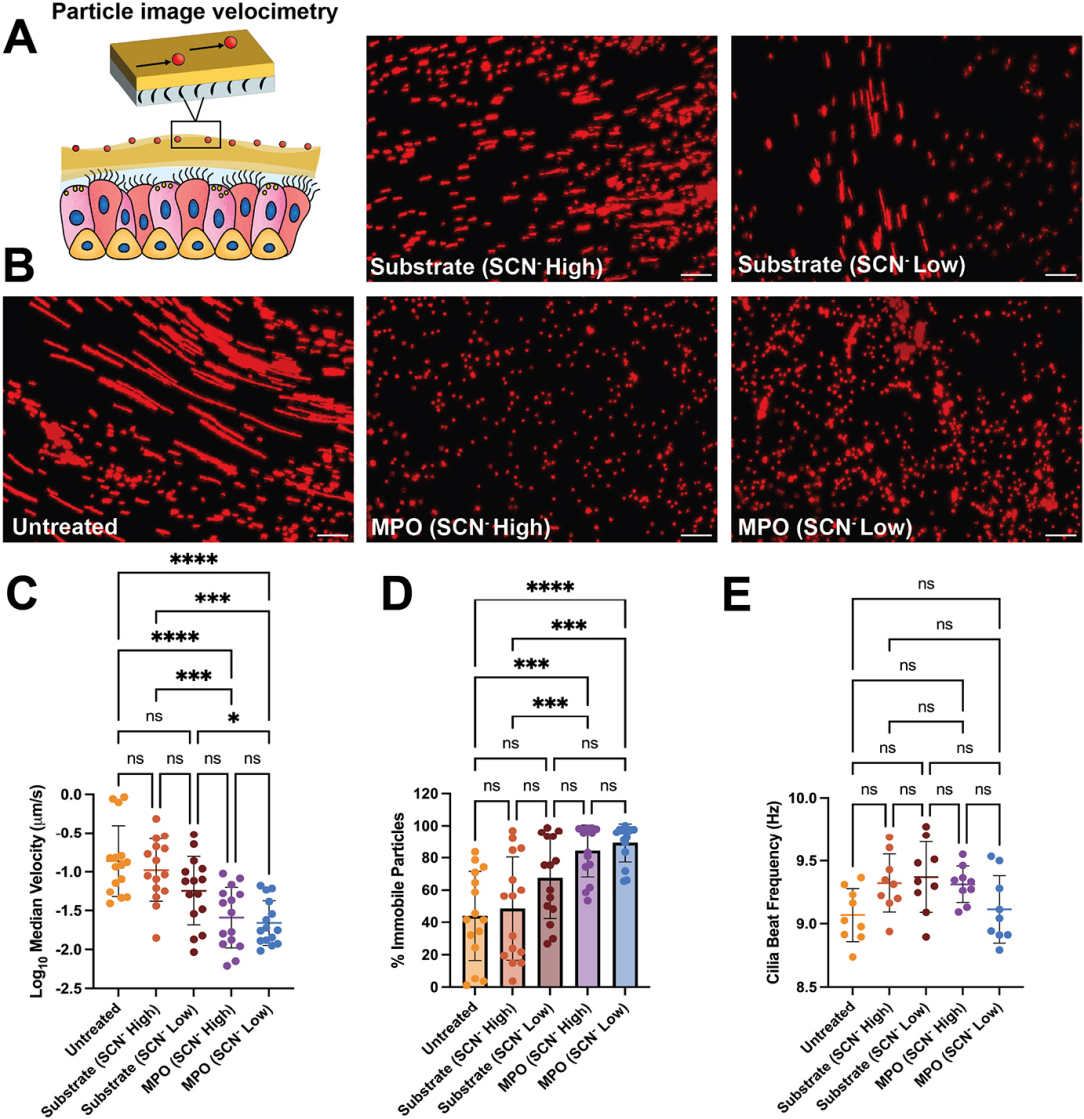
**MPO reduces MCT *in vitro*.** (A) Schematic showing the data collection method for evaluation of MCT using particle image velocimetry of fluorescent microspheres. (B) Maximum pixel intensity projections created from videos of microspheres undergoing MCT across the apical surface of cultures after treatment with MPO–substrate or substrate-only solutions for 24 h. Scale bars: 50 μm. (C) The velocities of each individual microsphere were calculated using a MATLAB algorithm, and log_10_ of the median velocity from each video was plotted. *n*=3 HAE cultures in each experimental group, with five videos in different regions analyzed for each individual culture. (D) The percentage of microspheres considered immobile in each video (percentage of microsphere trajectories with velocities under 0.1 μm/s). (E) Cilia beat frequency of the HAE cultures was quantified using both FIJI and MATLAB. *n*=3 HAE cultures in each experimental group, with three videos in different regions analyzed for each individual culture. In C-E, the statistical analyses performed were one-way ANOVAs with Tukey's multiple comparison tests (ns, no significance; *P<0.05, ***P<0.001, ****P<0.0001). Data in C-E display the mean±s.d.

Particle imaging velocimetry was performed using fluorescence video microscopy of microspheres to determine the efficiency of MCT after treatment with either the MPO–substrate or substrate-only solutions for 24 h (Fig. 2A). MCT velocity was reduced most significantly in both MPO–substrate treatment groups regardless of SCN^−^ concentration (Fig. 2B,C). Both the substrate-only solutions alone had modest effects on MCT velocity, although these were not significantly different from those of the untreated HAE cultures (Fig. 2B,C). Based on the calculated velocities, the percentage of immobile microspheres was also determined, where a velocity of 0.1 μm/s was considered immobile (Fig. 2D). This was chosen as the cut-off velocity because it meant that, within the 20 s of the video, the microsphere failed to travel at least the length of one particle radius from its starting position. An average of ∼60% of microspheres on the untreated control cultures were considered mobile, but the vast majority (∼85-90%) of microspheres became immobile on the MPO–substrate-treated cultures. To determine whether the drop in MCT was due to changes to the ciliary beating, brightfield video was used to quantify the cilia beat frequency (CBF). Higher concentrations of H_2_O_2_ are known to reduce CBF transiently; however, in this work, the H_2_O_2_ concentration applied to the HAE cells was 10 μM, far below what is reported to significantly impair CBF or cause irreversible ciliostasis (Burman and Martin, 1986; Kobayashi et al., 1992). No significant differences in the CBF between treatment groups 24 h after treatment were seen (Fig. 2E).

We also investigated the viability and ciliation of the HAE cultures using flow cytometry. Loss of ciliation of the airway epithelium can occur as a result of exposure to ROS (Mata et al., 2012). Cells were stained with a viability dye and an antibody to detect α-tubulin, a cilia marker. We found no significant alterations in the percentage of dead cells in the total cell population (Fig. S1A). Although the percentage of live ciliated cells was slightly decreased in all groups treated with ROS-containing solutions, the percentage of live ciliated cells in the total cell population did not exhibit major alterations (Fig. S1B) and was very similar to previous reports of percentage ciliation of BCI-NS1.1 cells (Walters et al., 2013). In addition, the mean fluorescence intensity of α-tubulin in the live α-tubulin-positive cell population was also not changed significantly (Fig. S1C), indicating that there were no large changes to the expression of cilia in the identified ciliated population. We also measured transepithelial electrical resistance (TEER) and found an average of ∼10% decrease in TEER 24 h after treatment of cultures with either of the MPO–substrate solutions and a ∼5% average decrease in substrate-only-treated cultures (Fig. S2). This aligns with previous reports that MPO treatment can reduce the TEER in HAE cell monolayers (Walters et al., 2013; Kim et al., 2008). The reduction in TEER seemed to indicate that the airway epithelium was still being affected by the MPO treatment despite the lack of changes to cell viability, ciliation and ciliary function of the HAE cultures. Therefore, we investigated the composition of the airway surface layer for differences that could account for the large drop in MCT velocity.

### MPO alters the viscosity and macromolecular composition of airway surface liquid

**Fig. 3.**
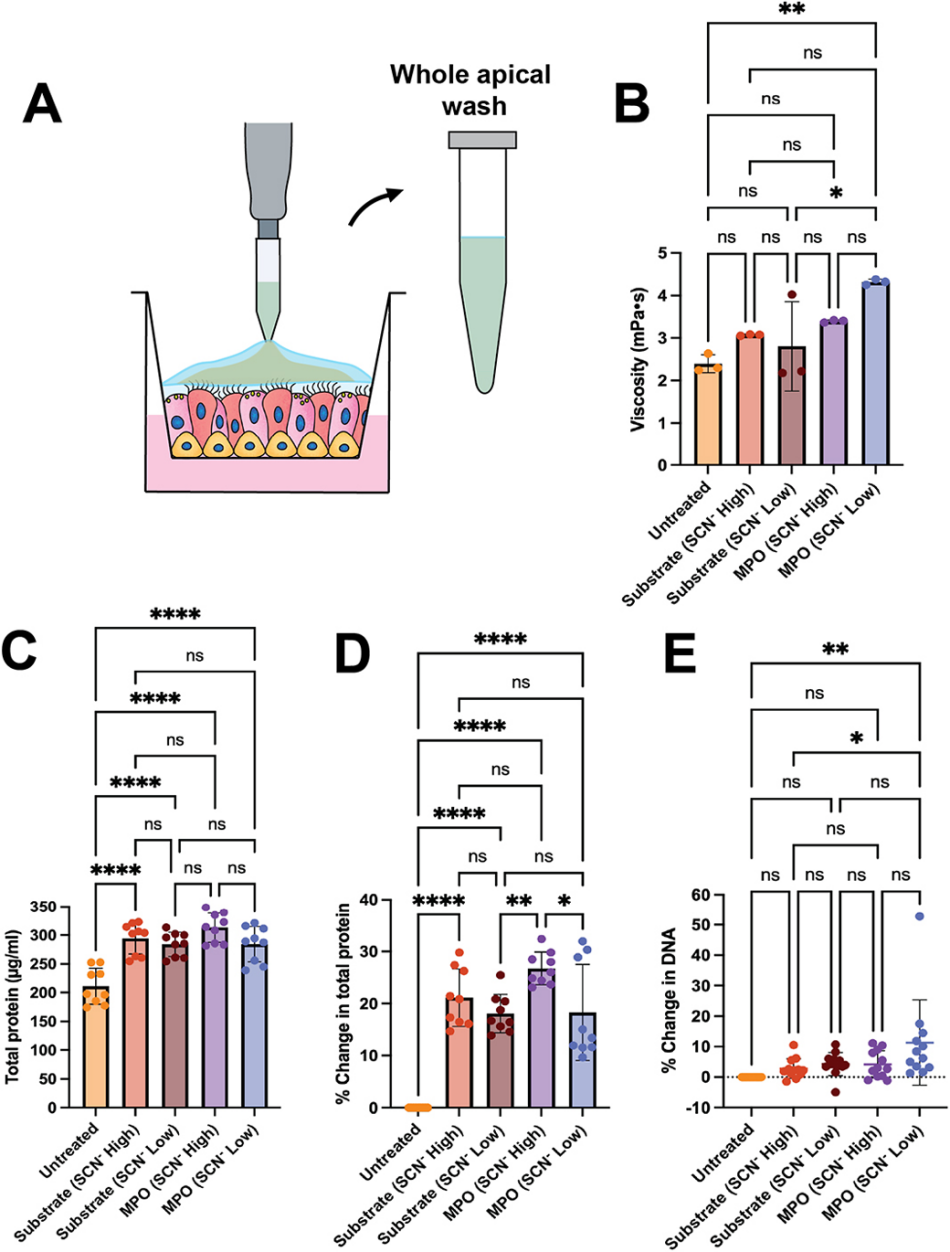
**MPO alters the viscosity and macromolecular composition of airway surface liquid.** (A) Schematic showing the collection of the whole apical wash from HAE cultures. (B) Viscosity of the apical wash samples from each treatment group quantified using a microviscometer. Three measurements of each sample were performed. The samples consisting of the pooled washes of two HAE cultures in each experimental group. (C) The total protein in the apical wash was quantified using a bicinchonic acid-based colorimetric reaction. Three independent experiments were performed, with the samples consisting of the pooled washes of two HAE cultures in each experimental group. Three technical replicates of each sample were included per experiment. (D) The concentration of total protein in each sample was determined using a standard curve of bovine serum albumin, and the percentage change in the absorbance values was calculated in relation to that of the untreated group. (E) The DNA in the apical wash was quantified using 3,5-diaminobenzoic acid (DABA) to produce a fluorescent reaction. The percentage change in fluorescence was calculated compared to that of the untreated group. Three independent experiments were performed, with the samples consisting of the pooled washes of two HAE cultures in each experimental group. Four technical replicates of each sample were included per experiment. Statistical significance in B-E was determined using one-way ANOVAs with Tukey's multiple comparison tests (ns, no significance; *P<0.05, **P<0.01, ****P<0.0001). Data in B-E display the mean±s.d.

The apical surfaces of the HAE cultures were washed with PBS following a 24-h incubation with either the MPO–substrate or substrate-only solutions, and the apical wash was collected (Fig. 3A). We measured the viscosity of the apical wash using a microviscometer and found that the MPO-substrate treated apical washings displayed the highest viscosity, suggesting that the macromolecular content was increased in these samples compared to that in the untreated samples (Fig. 3B). The MPO/SCN^−^ low treatment group had the highest viscosity. In addition, we determined that exogenous treatment of mucus harvested from HAE cultures with MPO did not significantly alter its viscosity (Fig. S3). To detect changes in the macromolecules produced in response to MPO exposure, the total protein and DNA content of the apical surface washes were quantified. The total protein in the whole apical wash of all the MPO–substrate- and substrate-only-treated cultures was significantly higher than that in the untreated cultures (Fig. 3C). In quantifying the percentage change in total protein compared to that in the untreated cultures, we found that there were significant increases for the SCN^−^ high substrate and MPO/SCN^−^ high conditions compared to the SCN^−^ low substrate and MPO/SCN^−^ low conditions (Fig. 3D). The DNA was found to be highest in the apical wash from the MPO/SCN^−^ low substrate treatment, with it being the only treatment to show statistical significance in the percentage change over the untreated condition (Fig. 3E). We confirmed this using another DNA staining method by adding Sytox Green to the apical wash samples (Fig. S4). The increase in DNA could indicate cytotoxicity in response to the upregulated production of HOCl. However, flow cytometry analysis of the HAE cells revealed no significant changes in the viability Fig. S1A. We also evaluated cellular viability based on lactose dehydrogenase (LDH) activity in the basolateral media from each individual culture 24 h pre-treatment and 1 h after treatment and calculated the percentage change (Fig. S5). The MPO/SCN^−^ low treatment group was the only one that exhibited increases in the percentage change in LDH activity above that of the untreated control. Both the increased DNA content in the airway surface layer and the minor increase in LDH activity suggest very-small and difficult-to-detect enhancements in cell death.

### MPO does not alter mucin expression or mucin–mucin crosslinking

**Fig. 4.**
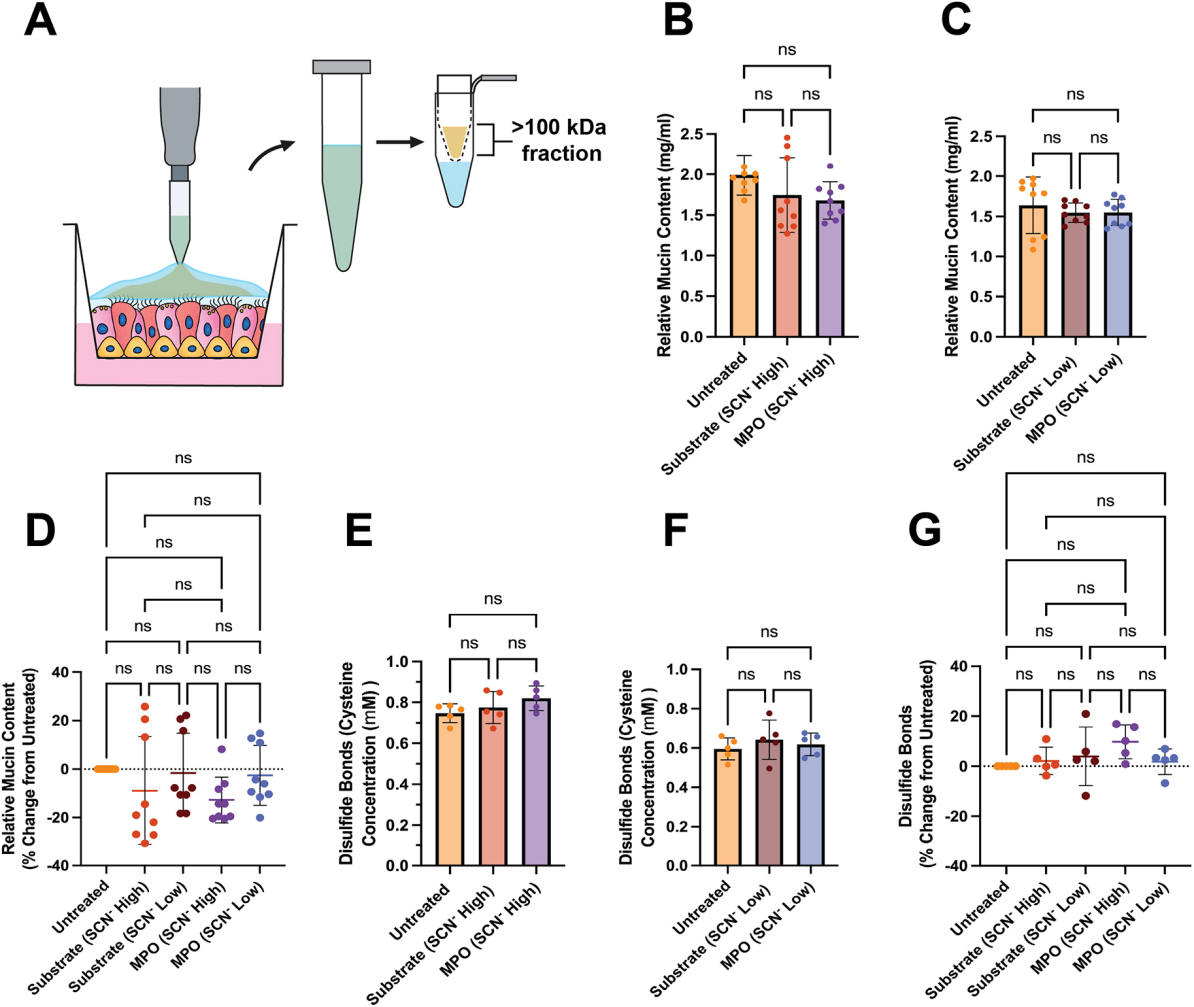
**MPO does not alter mucin expression or mucin–mucin crosslinking.** (A) Schematic showing the collection of the whole apical wash from HAE cultures and subsequent separation of the >100 kDa fraction using a centrifugal filter. (B,C) The relative mucin content of the >100 kDa fraction in the SCN^−^ high substrate-only and MPO-treated cultures (B) and the SCN^−^ low substrate-only and MPO-treated cultures (C) was assessed using a 2-cyanoacetamide-based reaction with O-linked glycans on mucins. Three independent experiments were performed, with the samples consisting of the pooled washes of two HAE cultures in each experimental group. Three technical replicates of each sample were included per experiment. (D) A standard curve of bovine submaxillary gland mucins was used to calculate the relative concentration of mucins in the samples, and the percentage change in fluorescence values was calculated in comparison to that of the untreated group. (E,F) The disulfide bonds in the >100 kDa fraction were quantified in the SCN^−^ high substrate-only and MPO-treated cultures (E) and the SCN^−^ low substrate-only and MPO-treated cultures (F) by blocking free cysteines, followed by treatment with a reducing agent to expose only the disulfide-bonded cysteines. Cysteines were detected fluorescently with monobromobimane. Five independent experiments were performed, with the samples consisting of the pooled washes of two HAE cultures in each experimental group. (G) The concentration of disulfide bonds in samples was determined using a standard curve of L-cysteine, and the percentage change in the fluorescence values was calculated in relation to that of the untreated group. Statistical significance in B-G was determined using one-way ANOVAs with Tukey's multiple comparison tests (ns, no significance). Data in B-G display the mean±s.d.

Based on the finding of upregulated protein expression in the apical wash, we investigated whether the treatments caused alterations to mucin glycoprotein expression and the network structure of mucus. It should be noted as well that a prior study characterized the effects of applying exogenous H_2_O_2_ to HAE cells on the expression of multiple mucin types in the airways and found that only *MUC5AC* mRNA expression was increased. However, the H_2_O_2_ concentrations tested were 10-100 times higher than those in this study (Kim et al., 2008). MUC5AC, when overexpressed, is generally known to form more viscoelastic mucus gels that inhibit MCT significantly (Song et al., 2022). Although MUC5AC is known to be the mucin typically upregulated by ROS and other neutrophil inflammatory mediators, MUC5B may also be upregulated. MUC5B, the other major secreted mucin in the airway, is known to be overexpressed in CF, often due to the cycle of ‘sterile inflammation’. Highly viscoelastic mucus causes necrosis of airway epithelial cells and leads to increased secretion of IL-1β, which induces secretion of MUC5B (Murphree-Terry et al., 2024). Considering this, we assessed the total mucin content of the airway surface layer after MPO–substrate or substrate-only treatment. To detect mucin glycoproteins in the airway surface layer, we took the whole apical wash from HAE cultures and isolated the >100 kDa fraction using centrifugal filtration (Fig. 4A). Mucins are very-large proteins (2-50 MDa) that will be retained in the >100 kDa fraction, whereas smaller glycoproteins will be removed (Song et al., 2020). We then took the >100 kDa fraction and fluorometrically detected O-linked glycosylation to evaluate mucin content (Fig. 4B-D). We found no significant differences, indicating no changes in mucin expression at the protein level. To ensure there were no changes at the mRNA level, we also evaluated the expression of *MUC5AC* mRNA using quantitative PCR. In agreement with the mucin detection assay, there were no significant changes found in *MUC5AC* mRNA expression (Fig. S6). In addition to mucin expression, we wanted to investigate changes to the microstructure of mucus. The microstructure of the mucus gel is directly influenced by disulfide bonding occurring between thiol groups in cysteine-rich regions of the mucin proteins. We quantified the disulfide bonds within the >100 kDa, mucin-containing fraction of the apical wash in HAE cells treated with the substrate or MPO–substrate solutions using a fluorometric assay (Fig. 4E-G). We found no significant differences between the disulfide bonds in groups, suggesting that MPO does not catalyze formation of additional mucin–mucin crosslinks at the concentrations tested in this study.

### MPO impairs MCT similarly to neutrophil elastase

**Fig. 5.**
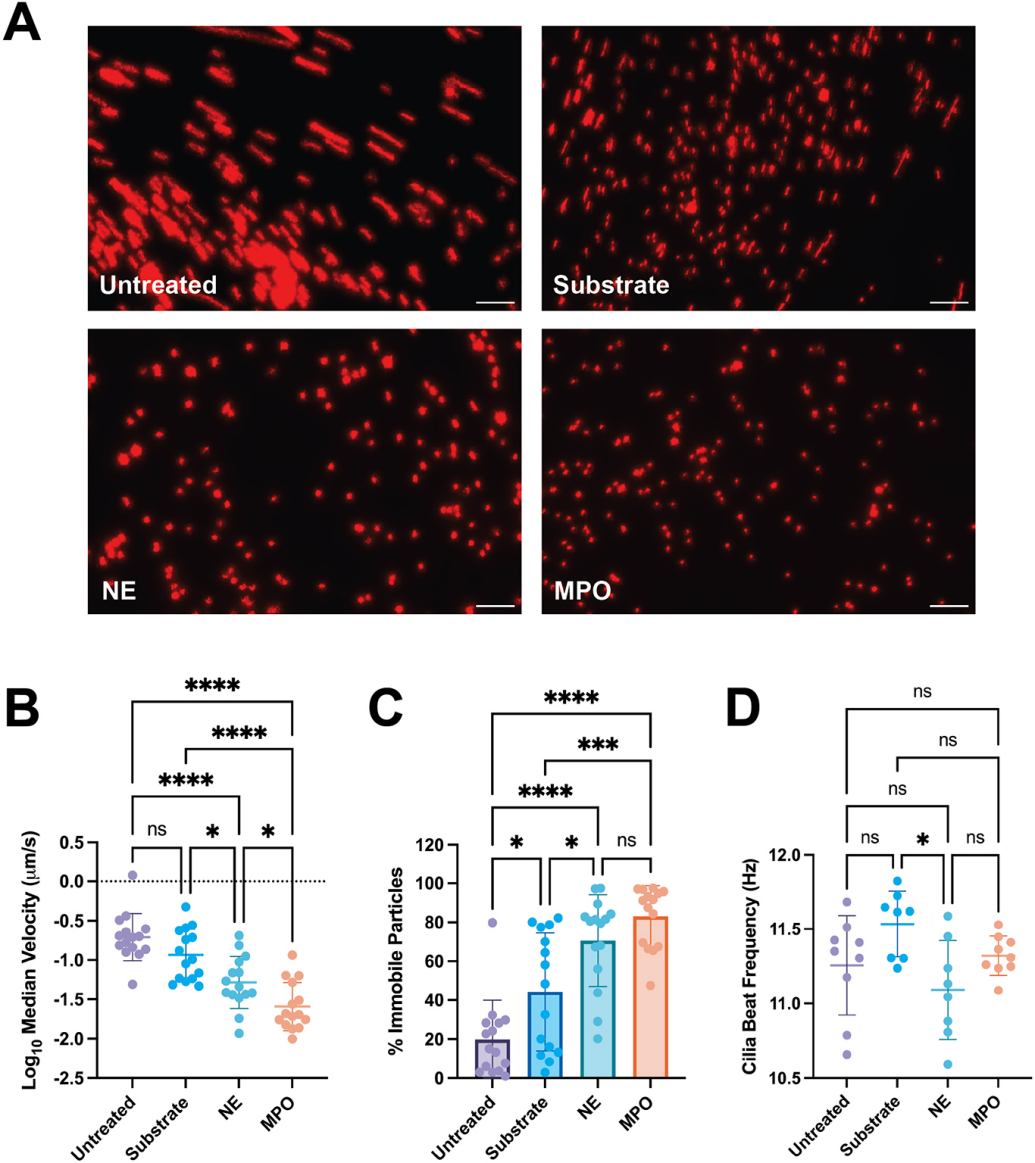
**MPO impairs MCT similarly to neutrophil elastase (NE).** (A) Maximum pixel intensity projections of microspheres being transported over the apical surface of HAE cultures after treatment with MPO–substrate, substrate-only or NE–substrate solutions for 24 h. Scale bars: 50 μm. (B) The MCT videos were analyzed with a MATLAB algorithm to determine the velocities of each microsphere, and the log_10_ of the median velocity for each video was plotted. *n*=3 HAE cultures in each experimental group, with five videos in different regions analyzed for each individual culture. (C) Microspheres were considered immobile if their velocity was less than 0.1 μm/s. The percentage of immobile particles was quantified and plotted for each video taken. (D) The cilia beat frequency of the HAE cultures was quantified using FIJI and MATLAB analyses. *n*=3 HAE cultures in each experimental group, with three videos in different regions analyzed for each individual culture. One-way ANOVAs with Tukey's multiple comparison tests were used to determine statistical significance in B-D (ns, no significance; *P<0.05, ***P<0.001, ****P<0.0001). Data in B-D display the mean±s.d.

We next compared the effects of MPO to neutrophil elastase (NE), another neutrophilic granular protein found in abnormally high concentrations in CF sputum. NE is a serine protease that has been widely studied and is well known to induce MUC5AC secretion by HAE cells (Kohri et al., 2002; Shao and Nadel, 2005; Fischer and Voynow, 2002). NE has been associated with worsening of lung function and airway clearance in CF (Dittrich et al., 2018; Mayer-Hamblett et al., 2007; Sagel et al., 2012; Sly et al., 2013). A previous study characterizing the effects of NE on MCT was done on anesthetized quails, in which there was a reduction in MCT over the course of 60 min after treatment with 100 μg/kg and 300 μg/kg NE (Tai et al., 1997). This was largely attributed to increased protein and DNA macromolecules found in the tracheal lavage fluid. In addition, aerosolized NE was shown to reduce MCT in ovine airways after 6 h (O'Riordan et al., 2012). One of the primary mechanisms by which NE likely impairs transport is by causing increased secretion of MUC5AC. MUC5AC overproduction is well known to reduce MCT (Song et al., 2022; Bonser et al., 2016). However, the effects of NE on MCT on human airway tissue cultures *in vitro* have not been directly characterized to the best of our knowledge. To compare the impact of MPO and NE on MCT, we added either 30 μg/ml MPO or 25 μg/ml NE, representative of the average concentrations of each found in CF sputum samples, to the SCN^−^ low substrate solution (Yuan et al., 2015; Dittrich et al., 2018; Sagel et al., 2001). By comparison, unaffected controls were found to have ≤0.1 μg/ml of MPO and NE in sputum samples (Yuan et al., 2015; Dittrich et al., 2018; Sagel et al., 2001). We found that groups with NE and MPO treatment showed significantly reduced MCT velocity compared to the untreated and SCN^−^ low substrate solution-treated groups alone (Fig. 5A,B). MPO treatment resulted in the lowest median MCT velocities, which were significantly different from those resulting from NE treatment, although the percentage of immobile microspheres was not significantly different between the NE- and MPO-treated cultures (Fig. 5B,C). We also evaluated the CBF and found similar results to the MCT experiments done in Fig. 2E. The substrate-treated cultures had a slightly increased CBF, whereas the NE-treated cultures had a lower CBF (Fig. 5D). This is consistent with some literature implying that NE can slow ciliary beating, especially at higher concentrations (Amitani et al., 2012).

### Impact of mucolytic agents on MCT in MPO-treated cultures

We sought to gain further insight into potential causes of MPO-mediated MCT dysfunction by evaluating changes in mucociliary function after treatment with mucolytic agents clinically used in the treatment of CF. Specifically, we applied MPO to HAE cultures and compared MCT velocity after treatment with N-acetylcysteine (NAC), hypertonic saline (HTS) or PBS as a control. NAC acts both as a reducing agent to disrupt disulfide bonds and as an antioxidant (Calzetta et al., 2018; Ehre et al., 2019). HTS at a concentration of 7% is commonly inhaled by CF patients to hydrate the mucus via osmotic pressure (Donaldson et al., 2006). Human cells exposed to MPO exhibit oxidative stress caused by oxidant acids, which can be ameliorated by the antioxidant peptide glutathione (Venglarik et al., 2003). NAC treatment enhances cellular production of glutathione (Calzetta et al., 2018). Considering this, we applied NAC in combination with MPO (NAC co-treatment) for 24 h to the mucosal surface of HAE cultures to determine whether oxidative stress caused by exposure to MPO could contribute to the impairment of MCT.

Another potential mechanism by which MPO could reduce MCT is via enhancements in disulfide bonding that would cause (1) increased mucus viscosity via increased mucin–mucin crosslinking and/or (2) mucus tethering to the epithelial surface of HAE cultures. Mucus tethering to the epithelium has been shown to be a major cause of MCT dysfunction in an *in vivo* piglet model of CF (Hoegger et al., 2014). Another study using IL-13-stimulated HAE cultures to model asthma *in vitro* found that treatment of HAE cells with a reducing agent reversed MCT dysfunction from mucus tethering (Bonser et al., 2016). It should be noted that tethered mucus would not be removed using the normal washing procedure to remove the secreted mucus layer from HAE cultures. Therefore, the experiments performed in Fig. 4 using the secreted mucus isolated from HAE washings would not likely account for mucus tethering. To determine whether mucus tethering and/or increased viscosity could be contributing factors to reduced MCT caused by MPO, we applied NAC to the mucosal surface of HAE cultures for 30 min following 24 h of treatment with MPO (NAC post-treatment). Mucus dehydration is another major cause of MCT impairment in CF, largely due to the malfunctioning of CFTR in transporting Cl^−^ ions into the airway surface liquid and upregulated activity of the epithelial Na^+^ channel (ENaC), causing hyperabsorption of Na^+^ ions from the airway surface liquid (Wark and McDonald, 2018). It is plausible MPO-mediated increases in mucin production could increase the concentration of mucus solids, thereby leading to dehydration at the airway surface. To test this, we applied HTS to the surface of HAE cultures for 30 min following 24 h of MPO treatment (HTS post-treatment) and quantified MCT.

**Fig. 6.**
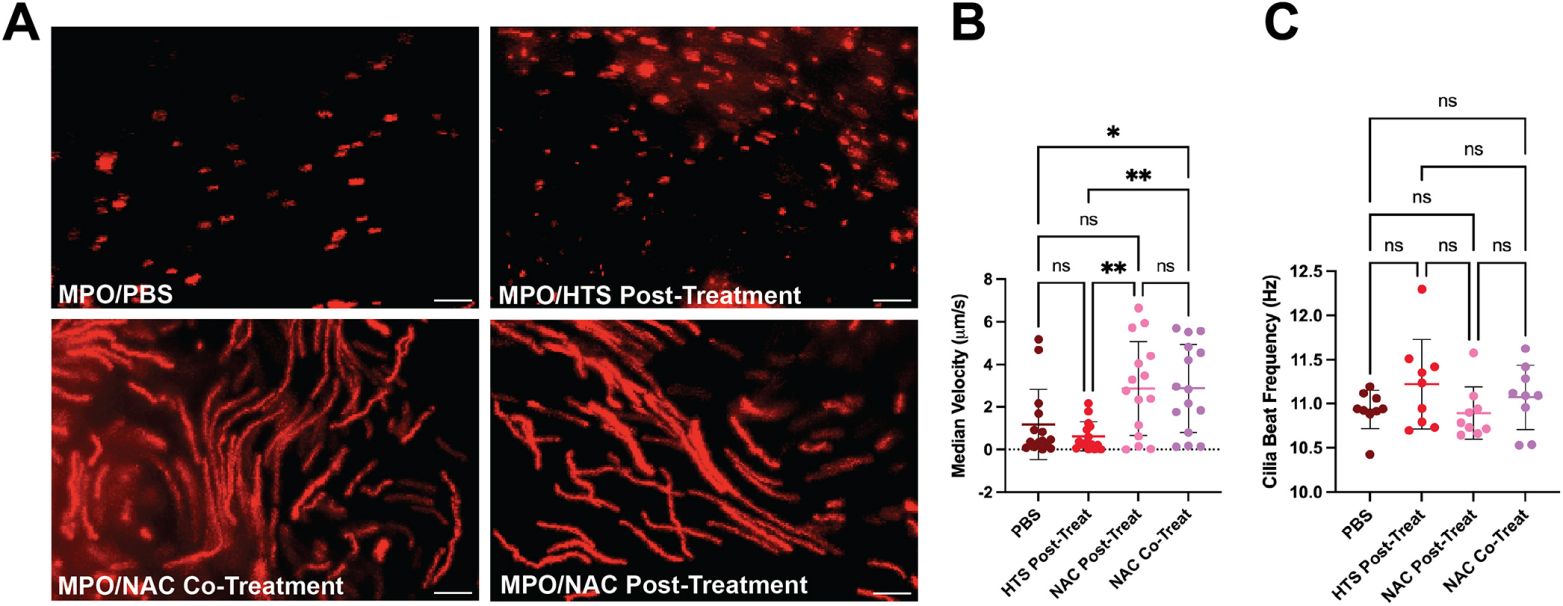
**N-acetylcysteine (NAC) treatment improves transport in human airway tissue cultures with MPO-induced mucostasis.** (A) Maximum pixel intensity projections of microspheres being transported over the apical surface of HAE cultures after treatment with MPO and/or NAC, hypertonic saline (HTS) or PBS. Scale bars: 50 μm. (B) The MCT videos were analyzed with a MATLAB algorithm to determine the velocities of each microsphere, and the median velocity for each video was plotted. *n*=3 HAE cultures in each experimental group, with five videos in different regions analyzed for each individual culture. (C) The cilia beat frequency of the HAE cultures was quantified using FIJI and MATLAB analyses. *n*=3 HAE cultures in each experimental group, with three videos in different regions analyzed for each individual culture. One-way ANOVAs with Tukey's multiple comparison tests were used to determine statistical significance in B and C (ns, no significance; *P<0.05, **P<0.01). Data in B and C display the mean±s.d.

We compared the MCT velocities of cultures exposed for 24 h to MPO and treated with NAC, HTS or PBS as a control (Fig. 6A-C). Volumes of solutions applied to the apical surface were kept equal among all groups. The ∼2.5-fold increase in MCT velocity with both NAC co-treatment and NAC post-treatment compared to PBS control treatment indicates that oxidative stress and mucus tethering to the epithelium are both potentially important mechanisms by which MPO impedes MCT (Fig. 6B). NAC is relatively weak, and our data are in alignment with a previous study in which 5 mM NAC treatment modestly enhanced the MCT velocity of CF HAE cultures by ∼2- to 3-fold (Ehre et al., 2019). HTS did not show efficacy in enhancing MCT velocity, signifying that dehydration is likely not a primary mechanism of MCT reduction by MPO. There were no significant changes in CBF (Fig. 6C). These data suggest that there are multiple mechanisms that contribute to the reduction in MCT caused by MPO treatment.

## DISCUSSION

We found that the application of exogenous MPO, likely to be at elevated levels owing to chronic inflammation in the CF lung, significantly slowed MCT in differentiated HAE cultures. It should be noted that excessive inflammation in the airways, primarily driven by neutrophils, remains a problem for many people with CF with or without access to modulator therapies. Recent studies have found that CFTR modulators are able to only partially dampen inflammation. One study found that inflammatory markers in CF sputum samples were reduced after ETI therapy, to levels very similar to those of people with non-CF bronchiectasis (Dittrich et al., 2018). Another recent study found that inflammatory markers in the sputum of CF patients taking ETI were decreased to levels similar to those found in samples from people with primary ciliary dyskinesia (Nussstein et al., 2025). In both cases, the sputum inflammatory markers in people with CF taking CFTR modulator drugs are decreased to levels comparable with those in people with other chronic lung diseases, yet still much higher than those in unaffected controls. Considering this, MPO-mediated defects in airway clearance due to neutrophilic inflammation could reduce lung function in individuals with CF independently of their use of highly effective CFTR modulators.

Within the airway surface layer in the CF-affected lung, the high extracellular concentrations and elevated enzymatic activity of MPO is due to increased neutrophil degranulation and NETosis. CF neutrophils have been shown to have an increased propensity to undergo degranulation compared to healthy neutrophils, releasing free MPO into the airway surface layer (Koller et al., 1995). In addition, very-high amounts of NETs are released into the mucus layer in CF (Marcos et al., 2015). Multiple studies have shown that MPO remains enzymatically inactive in NETs owing to being complexed with DNA (Worlitzsch et al., 1998; Parker et al., 2012). However, daily inhalation of DNase is one of the most widely used CF treatments to degrade the DNA ultrastructure of NETs and improve MCT efficiency (Cystic Fibrosis Foundation Patient Registry, 2025; Terlizzi et al., 2022). Previous research has found that DNase treatment of NETs causes the release of active MPO (Parker et al., 2012). Therefore, our results showing that MPO decreases MCT support the notion that there may be unintended effects of DNase treatment in releasing active NET-bound MPO.

Both Cl^−^ and SCN^−^ are transported via CFTR, and the balance of these ions causes MPO to generate different oxidant products. Without CFTR modulators, SCN^−^ concentrations in CF airways are very low, likely encouraging the production of the highly damaging oxidant HOCl (Kettle et al., 2004). It is plausible that people taking CFTR modulator therapies have concentrations of SCN^−^ restored to near normal levels and enhanced HOSCN production in the airways (Malkovskiy et al., 2019). However, we found that MCT is significantly impaired by MPO treatment independent of SCN^−^ concentration. After the 24-h treatment with MPO, we found no major changes in ciliation or CBF, suggesting that inhibition of MCT was caused by changes to the airway surface layer. After washing the apical surface layer off each of the cultures and measuring the viscosity of each, we found that they were highest in the MPO-treated cultures. Specifically, the MPO/SCN^−^ low substrate solution condition had the highest viscosity. Therefore, we explored the macromolecular composition of the apical surface layer of each treatment group. We found that MPO in combination with both SCN^−^ high substrate solution resulted in the highest protein content in the airway surface layer. It is unlikely that the apparent increase in concentration is due to the exogenous addition of MPO alone, as we estimate that this would yield <1% increase in protein given the total effective dose of MPO being 0.6 μg per culture. Therefore, increases in protein content of airway surface liquid following MPO–substrate and substrate-only treatments are presumably due to elevated secreted protein concentrations.

In addition to changes in protein content of the mucus, treatment with MPO and SCN^−^ low substrate in combination led to the highest increases in DNA content in the apical surface. This could be because of the increased production of HOCl, which is known to be more cytotoxic than HOSCN. However, the mucus layer does seem to be protective against oxidative stress, as we did not find that our MPO–substrate treatments were highly cytotoxic. This is in agreement with prior studies that have shown that the components of the extracellular matrix and CF mucus interact strongly with MPO, likely owing to the interaction of cationic MPO with anionic macromolecules such as DNA, glycosaminoglycans and mucins (Worlitzsch et al., 1998; Van der Vliet et al., 2000; Vanichkitrungruang et al., 2020; Cai et al., 2020). Multiple studies have concluded that MPO often reacts primarily with the components of CF mucus rather than the underlying cells. In one study, Chinese hamster ovary cells were treated with 66 μM MPO and 300 μM H_2_O_2_, which caused ∼80% of cells to die. When heat-inactivated CF sputum was added to the media at a 1:20 dilution and treated again with MPO and H_2_O_2_, the cytotoxicity dropped to ∼0% (Worlitzsch et al., 1998). This is supported by other research that conducted analysis of proteins in CF sputum and bronchoalveolar lavage samples and found hallmarks of HOCl-mediated oxidation (Kettle et al., 2004; Van der Vliet et al., 2000). The antioxidant glutathione is also secreted by the airway epithelium to scavenge oxidants in the mucus. A previous study using 16HBE14o- cells mounted in an Ussing chamber found that the addition of physiologically relevant concentrations of glutathione completely prevented HOCl-induced disturbances in the TEER (Venglarik et al., 2003). Therefore, it is plausible that we could not detect major differences in viability. Although we did not see large changes in the cytotoxicity of our MPO–substrate treatments, we still believe that the elevated DNA found in the apical surface layer is from minute increases in cell death that are not easily detectable. DNA is a large macromolecule, and even slight increases in cell death can contribute largely to enhanced mucus viscoelasticity (Boboltz et al., 2023).

Given the increase in protein content of the apical wash and the ability of many inflammatory mediators to induce mucin protein secretion in CF, we also evaluated mucin expression after MPO treatment. However, we found no upregulation of mRNA or protein expression for mucins. In addition, we assessed the formation of disulfide bonds between mucins after MPO treatment given that HOCl can oxidize thiol groups. We did not find any changes in disulfide bond formation between mucins, indicating that the increased viscosity of mucus was not due to increased mucin crosslinking. Similar peroxidase enzymes secreted by the airway epithelium, thyroid peroxidase (TPO) and lactoperoxidase (LPO), were shown to enhance disulfide bonding between thiolated polymers (Liegeois et al., 2024). In these experiments, the mucus layer of HAE cultures was completely removed using multiple dithiothreitol and PBS washes, and a solution of a thiolated hyaluronic acid polymer was overlaid on the apical surface of the HAE cultures. Conversely, we found no evidence that MPO treatment alters mucin expression or crosslinking. It should be noted that HAE cells express other proteins with antioxidant activity, such as glutathione peroxidase, catalase, glutathione S-transferase, peroxiredoxin or thioredoxin, which may have had a protective effect and limited MPO-mediated changes to mucin content or cross-linking (Wright et al., 1994; Wark and McDonald, 2018; Nussstein et al., 2025). As such, we speculate that MPO-mediated deficits in MCT could, at least in part, be a result of disrupted epithelial barrier integrity, as evidenced by changes in TEER, which may cause fluid and ion imbalance, leading to dehydration of the airway surface liquid layer. This could, in effect, increase the concentration of mucin and other apically secreted proteins without having a direct impact on the extent of mucin glycoprotein expression.

We also compared the effects of MPO on MCT to another neutrophilic granular protein, NE, that is very well characterized to cause deficits in MCT, which can be attributed at least in part to the ability of NE to induce MUC5AC secretion (Kohri et al., 2002; Shao and Nadel, 2005; Fischer and Voynow, 2002). We applied MPO and NE at concentrations similar to their reported average concentrations in CF sputum samples, 30 μg/ml and 25 μg/ml, respectively (Yuan et al., 2015; Dittrich et al., 2018; Sagel et al., 2001). We found that MCT was significantly impaired by both NE and MPO, with MPO-treated cultures having the lowest MCT velocities compared to those of NE-treated cultures. One contributing factor to this result may be that NE is a protease that cleaves various proteins in the airway surface layer, potentially loosening the mucus gel network and enhancing MCT. Overall, our data indicate that both MPO and NE are important NET-associated components to consider in CF and other chronic lung conditions in which mucociliary clearance is impaired. We also found that the impact of MPO on MCT could be mitigated using NAC but not HTS. This result offered additional insight into potential mechanisms by which MPO impairs MCT. The improvement in MCT when HAE cultures were treated with NAC in combination with MPO for 24 h and following 24 h of MPO exposure suggests that oxidative stress of the airway epithelium, enhanced mucus viscosity and mucus tethering are potential mechanisms of MPO-mediated MCT dysfunction. By comparison, we do not believe that MPO contributes significantly to mucus dehydration given that there was no improvement in MCT following HTS treatment of MPO-exposed HAE cultures. We believe that there are several mechanisms by which MPO affects the airway epithelium to alter mucociliary function, and further investigation is needed to firmly establish the primary causes. Our data indicate that implementation of these reducing agents could potentially prove useful in improving MCT, especially in individuals with high levels of MPO in the airways due to neutrophilic inflammation.

This work establishes that MPO can directly impair MCT *in vitro* via cell-mediated changes to airway surface liquid properties. Adjusting the SCN^−^ concentrations to generate primarily HOSCN over HOCl did not make substantial differences in MCT. Treatment with MPO resulted in a similar decrease in MCT to that with treatment with NE, another granular protein found to be upregulated in CF airways and known to cause deficits in airway clearance function. We also acknowledge the limitations of this study, including the use of a BCI NS1.1 immortalized normal HAE cell line instead of primary CF cultures. In addition, we did not consider the use in our experiments of co-treatment with hypertonic saline, which is inhaled daily by many people with CF and can raise the amount of Cl^−^ in the airways. This may boost HOCl production even further and contribute to more damage. In addition, different fluorometric assays were used to investigate changes in DNA, mucin glycosylation and disulfide bonds. Fluorometric assays have limitations as the fluorescence can be affected by changes in reagent pH or temperature, interference from biological samples and accidental light exposure, which can cause photobleaching. These data show MPO as a potential therapeutic target to improve MCT and further reduce mucus plugging in people with CF both receiving and not receiving CFTR modulator therapies. The MPO-mediated reduction in MCT could also apply to similar lung pathologies with neutrophil-driven inflammation such as non-CF bronchiectasis, chronic obstructive pulmonary disease or acute infections.

## MATERIALS AND METHODS

Additional information on the methods used to collect supplementary figure data can be found in the Supplementary Materials and Methods.

### ALI culture of human airway epithelial cells

The h-TERT immortalized human airway epithelial basal cell line, BCI-NS1.1 (provided by Ronald Crystal, Cornell University, Ithaca, NY, USA), was cultured in a flask in Pneumacult Ex-Plus Medium (Stemcell Technologies) until cells reached 70-80% confluency. To establish ALI cultures, cells were seeded at a density of 104 cells/cm^2^ on collagen-coated 6.5 mm Transwell cell culture inserts (Corning) with Pneumacult Ex-Plus Medium added to both the apical and basolateral compartments. After the cells formed a confluent monolayer, the medium was removed from the apical compartment to expose cells to the air. The medium in the basolateral compartment was also switched to Pneumacult ALI (Stemcell Technologies) once airlifted. The cells were cultured at the ALI for at least 28 days to ensure full differentiation of the epithelium, and medium was changed every 2 days. After reaching maturity, mucus was washed from the surface of the cells at least once per week by adding 250 μl DPBS (without Ca^+^ and Mg^2+^) to the apical compartment. The cells were incubated with the PBS for 30 min at 37°C and 5% CO_2_, then the PBS was removed by aspirating or by collecting with a pipette if using the mucus washings.

### MPO/NE/substrate solution treatment of HAE cultures

All HAE cells were washed 24 h prior to treatment with MPO, NE or substrate solutions. Potassium thiocyanate (KSCN) and sodium chloride (NaCl) were dissolved in sterile ultrapure water. H_2_O_2_ was diluted in sterile ultrapure water. The NaCl, KSCN and H_2_O_2_ solutions were combined into one solution and sterile filtered. MPO from human neutrophils (Athens Research and Technology) or a vehicle (substrate only) was added to each solution to achieve a final concentration of 500 μM KSCN, 90 mM NaCl and 10 μM H_2_O_2_ in the SCN^−^ high substrate solution and 45.45 μM KSCN, 90 mM NaCl and 10 μM H_2_O_2_ in the SCN^−^ low substrate solution. In solutions containing MPO, the final concentration of MPO was 30 μg/ml. In solutions containing NE from human neutrophils (Athens Research and Technology), the final concentration of NE was 25 μg/ml. Once the solutions were prepared, fully differentiated HAE cells were treated apically with 20 μl of each mixture for 24 h. During the 24-h incubation period, the 20 μl of liquid applied to the surface of the cultures was completely absorbed.

### MCT quantification

All HAE cultures were washed and treated with MPO, NE or substrate solutions as described above. MCT was quantified using particle image velocimetry data collection and analysis methods described previously (Corkran et al., 2025). 2 μm red fluorescent microspheres (Thermo Fisher Scientific FluoSpheres) were sonicated for 10 min, then diluted 1:3000 in sterile PBS and mixed thoroughly. 20 μl of the microsphere solution was applied apically to the mucosal layer of the cells. Cultures were incubated with the microspheres for 15 min (Figs 2 and 5) or 30 min (Fig. 6) to allow mucolytic agents to take effect at 37°C, 5% CO_2_. After this incubation, the cultures were removed and placed in a six-well plate using tweezers so that the cell culture insert membrane was flush with the bottom of the six-well plate and could be imaged clearly. This process of applying the microspheres, incubating and moving the cultures was repeated for each batch of three cultures in each treatment group immediately prior to imaging. The apical surfaces of the cultures were imaged using fluorescence video microscopy. Videos of microspheres undergoing MCT in five random regions of each HAE culture were taken at 10× magnification for 20 s at an exposure time of 150 ms. Each video was analyzed to quantify the velocity of the microspheres using MATLAB. The MATLAB analysis identified and tracked the trajectories of the microspheres, then computed the velocity of each based on the *x*- and *y*-coordinates of the microspheres’ trajectory. The median MCT velocity from each video was plotted.

### Treatment of HAE cultures with mucolytic agents

All HAE cultures were washed with PBS 24 h prior to treatment with MPO in the SCN^−^ low substrate solution, prepared in the same manner as described directly above. In HAE cultures that received a NAC co-treatment, NAC (5 mM final concentration) was added to the SCN^−^ low substrate solution and sterile filtered before adding 30 μg/ml MPO. Then, 20 μl of the MPO/NAC solution was added to the surface and incubated for 24 h. The next day, 20 μl of PBS containing microspheres was added to the apical surface for 30 min, and measurement of MCT was performed. All other cultures received the same treatment of 20 μl of MPO in SCN^−^ low substrate solution for 24 h. In cultures receiving HTS post-MPO treatment, 20 μl of HTS containing microspheres was applied to the apical surface and incubated for 30 min prior to measuring MCT. In cultures that received the NAC post-MPO treatment, 20 μl of PBS containing both 5 mM NAC and microspheres was added for 30 min prior to evaluating MCT. In control cultures with no mucolytic applied, 20 μl of PBS containing microspheres was added to the apical surface and incubated for 30 min before MCT evaluation.

### CBF quantification

CBF was quantified using analysis of brightfield microscopy videos using previously disseminated methods (Corkran et al., 2025). Directly after taking the MCT videos, the microscope settings were switched to brightfield. The focus on the epithelium was adjusted until ciliary beating was clearly visible. Videos were taken in three random areas of each HAE culture using a 10× objective for 10 s at 20 ms. CBF was quantified using FIJI and MATLAB to analyze each video. Briefly, the mean intensity over the time of the video was obtained in FIJI in three randomly selected regions of each video. The mean intensity versus time data were saved and analyzed using a MATLAB script. The MATLAB script identified the local maxima that represent the beating of cilia in the mean intensity versus time data. The number of local maxima over the time was computed and yielded the CBF of each of the three regions selected from each individual video. The mean CBF value from each video was plotted.

### Protein quantification in HAE apical wash

The total protein in the apical wash of HAE cells was determined using a bicinchonic acid (BCA) assay (Thermo Fisher Scientific), according to kit instructions. HAE cells were washed according to the methods described above following 24 h of treatment with MPO–substrate or substrate-only solutions. The whole apical wash collected from two cultures in each treatment group were pooled and thoroughly mixed. A standard curve was prepared using dilutions of bovine serum albumin, and 25 μl of samples and standards were added to a clear 96-well plate. 200 μl of the protein detection reagent was added to the wells and incubated at 37°C for 30 min to allow the colorimetric reaction to take place. The absorbance was read on a plate reader at a wavelength of 562 nm.

### DNA quantification in HAE apical wash

The total DNA in the apical wash of the HAE cells was determined using 3,5-diaminobenzoic acid (DABA), which produces fluorescent compound when reacting with the aldehydes in DNA (Duncan et al., 2016). The whole HAE apical washes from two cultures in each treatment group were pooled and thoroughly mixed. 30 μl of the apical wash samples were mixed with 30 μl of 20% w/v DABA and incubated for 1 h at 60°C on a heat block. The reaction was quenched with 1 ml of 1 M HCl and mixed. Samples were added to the wells of black 96-well plates, and the fluorescence was read at excitation/emission wavelengths of 340/420 nm (Duncan et al., 2016).

### Viscosity quantification of apical wash

After 24 h of treatment, the whole apical washes from two cultures in each treatment group were collected, pooled and mixed thoroughly. The viscosity of the apical wash samples from each condition were quantified at room temperature using a Rheosense microviscometer with an A05 chip installed. A viscosity standard of 10 mPa•s was tested prior to testing samples to ensure that the microviscometer was properly functioning. Prior to testing samples, the microviscometer was run on cleaning mode twice with deionized water. The microviscometer pipette was loaded with ∼400 μl of the apical wash samples, ensuring that any air bubbles were removed. The viscosity was measured using the automatic mode, which determines the optimal parameters for microviscometer sample priming and measurements including the volume and shear/flow rate.

### Mucin content assay

The whole apical wash was collected from two HAE cultures in each treatment group and mixed thoroughly. 450 μl of the whole apical wash was added to a 0.5 ml 100 kDa molecular mass cut-off Amicon Ultra centrifugal filter (Sigma-Aldrich) and centrifuged for 20 min at 14,000 ***g***. The centrifugal filter was flipped upside down into a new microcentrifuge tube, and the contents was removed by centrifuging for 2 min at 2000 ***g*** to yield the >100 kDa fraction of the HAE apical wash. The mucin content of the samples was evaluated using a previously established fluorometric assay to detect the O-linked glycans of glycoproteins (Crowther and Wetmore, 1987). Briefly, a standard curve of bovine submaxillary gland mucins (Sigma-Aldrich) was made using serial dilutions of a 2 mg/ml stock in PBS. A solution of 0.6 M 2-cyanoacetamide (CNA) was mixed with a solution of 0.15 M sodium hydroxide (NaOH) to achieve final concentrations of 0.1 M CNA and 0.025 M NaOH. 60 μl of this reagent was added to 30 μl of each sample and incubated on a heat block at 100°C for 30 min. The tubes were centrifuged briefly using a tabletop centrifuge before quenching the reaction. The reaction was quenched by adding 500 μl of a buffer consisting of 0.6 M boric acid with a pH of 8.0 to each sample. The samples were added to a black 96-well plate, and the fluorescence was quantified using a plate reader with excitation/emission wavelengths of 336/383 nm.

### Disulfide bond quantification

The whole apical wash was collected from two HAE cultures from each treatment group after 24 h and mixed thoroughly. 450 μl of the whole apical wash was added to a 0.5 ml 100 kDa molecular mass cut-off Amicon ultracentrifuge filter (Sigma-Aldrich) and centrifuged for 20 min at 14,000 ***g***. The disulfide bonds were quantified using a previously established fluorometric assay (Yuan et al., 2015). Briefly, 50 μl of samples were aliquoted, and 8 M guanidine-HCl was added to achieve a final volume of 500 μl. The diluted samples were treated with 10% v/v 500 mM iodoacetamide (final concentration of 50 mM) for 1 h at room temperature to block free cysteines. Samples were then treated with 10% v/v 1 M dithiothreitol (DTT) at 37°C for 2 h to reduce disulfide bonds. Using 7 kDa molecular mass cut-off Zeba desalting columns according to the manufacturer's instructions (Thermo Fisher Scientific), small molecules were removed from the samples, and buffer exchange was performed with 50 mM Tris-HCl pH 8.0. A standard curve of L-cysteine was made using serial dilution of a 2.5 mM stock in Tris-HCl buffer. 70 μl of each sample or standard was loaded into black 96-well plates, then an equal volume of 2 mM monobromobimane (Cayman Chemical) was added to each well to fluorescently detect cysteine residues. The plate was incubated for 15 min covered at room temperature, then fluorescence was quantified using a plate reader with an excitation/emission wavelength of 395/490 nm.

### Statistical analysis

All graphing and statistical analyses were performed using GraphPad Prism 11. One-way ANOVAs with Tukey's multiple comparisons test post hoc were performed to compare experimental groups. *P*<0.05 was considered statistically significant.

## Supplementary Material

10.1242/dmm.052764_sup1 Supplementary information
